# The Characteristic Recovery Time as a Novel, Noninvasive Metric for Assessing *In Vivo* Cartilage Mechanical Function

**DOI:** 10.1007/s10439-020-02558-1

**Published:** 2020-07-14

**Authors:** Hattie C. Cutcliffe, Keithara M. Davis, Charles E. Spritzer, Louis DeFrate

**Affiliations:** 1 Department of Orthopaedic Surgery, Duke University School of Medicine, Durham, NC USA; 2 Department of Biomedical Engineering, Duke University, Durham, NC USA; 3 Department of Radiology, Duke University School of Medicine, Durham, NC USA; 4 Department of Mechanical Engineering and Materials Science, Duke University, Durham, NC USA

**Keywords:** Osteoarthritis (OA), Magnetic resonance imaging (MRI), Quantitative MRI (qMRI), Cartilage, Recovery, T1rho mapping, T2 mapping

## Abstract

Osteoarthritis (OA) is a disease characterized by the degeneration of cartilage tissue, and is a leading cause of disability in the United States. The clinical diagnosis of OA includes the presence of pain and radiographic imaging findings, which typically do not present until advanced stages of the disease when treatment is difficult. Therefore, identifying new methods of OA detection that are sensitive to earlier pathological changes in cartilage, which may be addressed prior to the development of irreversible OA, is critical for improving OA treatment. A potentially promising avenue for developing early detection methods involves measuring the tissue’s *in vivo* mechanical response to loading, as changes in mechanical function are commonly observed in *ex vivo* studies of early OA. However, thus far the mechanical function of cartilage has not been widely assessed *in vivo*. Therefore, the purpose of this study was to develop a novel methodology that can be used to measure an *in vivo* mechanical property of cartilage: the characteristic recovery time. Specifically, in this study we quantified the characteristic recovery time of cartilage thickness after exercise in relatively young subjects with asymptomatic cartilage. Additionally, we measured baseline cartilage thickness and T1rho and T2 relaxation times (quantitative MRI) prior to exercise in these subjects to assess whether baseline MRI measures are predictive of the characteristic recovery time, to understand whether or not the characteristic recovery time provides independent information about cartilage’s mechanical state. Our results show that the mean recovery strain response across subjects was well-characterized by an exponential approach with a characteristic time of 25.2 min, similar to literature values of human characteristic times measured *ex vivo*. Further, we were unable to detect a statistically significant linear relationship between the characteristic recovery time and the baseline metrics measured here (T1rho relaxation time, T2 relaxation time, and cartilage thickness). This might suggest that the characteristic recovery time has the potential to provide additional information about the mechanical state of cartilage not captured by these baseline MRI metrics. Importantly, this study presents a noninvasive methodology for quantifying the characteristic recovery time, an *in vivo* mechanical property of cartilage. As mechanical response may be indicative of cartilage health, this study underscores the need for future studies investigating the characteristic recovery time and *in vivo* cartilage mechanical response at various stages of OA.

## Introduction

Osteoarthritis (OA) is a debilitating disease affecting articular cartilage, the tissue lining the ends of long bones in synovial joints.^33^ As the clinical diagnosis of OA requires the existence of radiographic imaging findings and pain, which typically do not present until advanced stages of OA,^32^ identification of new detection methods that are sensitive to earlier pathological changes in cartilage is critical for improving OA treatment. Importantly, several changes may occur in cartilage tissue during OA before clinical signs, such as pain and radiographic abnormalities, are present.^32^ These include altered cartilage composition, such as decreases in proteoglycan content, changes in collagen content and organization, and changes in water content.^20^,^22^,^32^,^37^,^43^ The mechanical response of cartilage to loading is also affected, as decreased stiffness and changes in permeability are observed in OA cartilage.^20^,^24^,^37^,^39^ Alterations in mechanical response may further the pathological cascade,^19^ as cartilage metabolism has been shown to be affected by its mechanical environment.^7^,^27^,^28^,^38^ As a result, measurement of changes in cartilage mechanical response may indicate OA pathology prior to the presence of radiographic features or pain,^24^ and may help identify those at risk of developing OA.

While assessment of mechanical response is classically performed in the *ex vivo* environment, magnetic resonance imaging (MRI) combined with loading has been used to quantify cartilage mechanical response *in vivo*.^9^,^36^ Under load (such as from exercise), water is forced out of the extracellular matrix. As this flow of water occurs over time, it gives rise to a time-dependent deformation of the cartilage.^34^ MRI can then be used to measure cartilage morphology before and after the exercise-induced flow of water, enabling measurement of cartilage deformation due to loading.^14^–^16^ For example, in recent work from our lab,^36^ tibial cartilage was mechanically loaded *in vivo* through repeated bouts of walking at different durations with MRI taken before and after each walk, allowing for a dose-dependent measurement of cartilage strain. *In vivo* strain was found to increase monotonically with walk duration and did so in a nonlinear, exponential-approach manner,^36^ consistent with *ex vivo* observations of cartilage creep.^34^

As the flow of water back into the tissue upon unloading is also time-dependent, the reversal of strain during unloading can similarly be quantified using MRI. Indeed, recent *ex vivo* work by our group^12^ showed that measuring the unloading response, or the recovery after loading, results in similar mechanical property characterization (aggregate modulus and characteristic time) as measuring the loading response, as is traditionally done. Not only would assessing cartilage recovery *in vivo* complement our prior work that investigated cartilage creep *in vivo*,^36^ it would also reduce MRI and analysis time, as only one pre-activity MRI and one bout of activity is necessary (compared to multiple bouts of activity and multiple pre/post MRI when measuring the creep response). Therefore, the purpose of this study was to develop a novel MRI methodology that can be used to noninvasively measure an *in vivo* mechanical property of cartilage: the characteristic recovery time. Specifically, our goal was to measure the mechanical response of tibial cartilage during recovery after loading to quantify tibial cartilage’s characteristic recovery time *in vivo*. Based on previous work from our lab,^12^ we hypothesized that tibial cartilage would exhibit a time-dependent recovery characterized by exponential approach, and that the characteristic time of this *in vivo* recovery would be comparable to literature values of characteristic times measured *ex vivo*. As the characteristic recovery time is a mechanical property of cartilage tissue that is related to cartilage’s thickness, stiffness, and permeability, we propose it here as a novel *in vivo* metric for describing cartilage mechanical function.

Moreover, quantitative MRI (qMRI) techniques have emerged as methods that reflect cartilage composition and health.^11^,^30^,^31^ Specifically, qMRI measures such as T1rho and T2 relaxation times are sensitive to changes in proteoglycan concentration, collagen content and organization, and water content,^11^,^18^,^30^,^45^ and can be obtained noninvasively. Studies have also shown that these qMRI measures change during OA progression,^20^,^28^–^31^,^35^ motivating their use as indicators of cartilage health. Therefore, baseline qMRI biomarkers of cartilage composition (T1rho and T2 relaxation times) and baseline cartilage thickness were also measured in this study to explore their potential relationships with the characteristic recovery time, to understand whether or not the characteristic recovery time provides additional information about cartilage that is not captured by these baseline qMRI metrics. As the characteristic recovery time reflects cartilage’s mechanical function, we hypothesized that it would not be significantly correlated with qMRI measures indicative of cartilage’s composition or with baseline cartilage thickness.

## Methods

### Data Collection

All study procedures were approved by the Duke University Health System Institutional Review Board (IRB). Eleven relatively young, asymptomatic subjects (5 male/6 female; mean age: 25 years, range: 22–32 years; mean BMI: 22.1 kg/m^2^, range: 19.8–24.7 kg/m^2^) provided informed written consent prior to their participation. Inclusion criteria were an age of 18–40 years and a normal BMI (18.5–24.9 kg/m^2^)^46^ while exclusion criteria were any history of lower extremity injury, any history of smoking, and any evidence of knee OA as assessed by MRI by an experienced musculoskeletal radiologist. As our prior work investigating creep *in vivo* examined ten subjects and found a significant correlation between loading and cartilage strain (strain increased with increasing walk duration),^36^ our goal was to analyze a similar number of subjects in the current study to investigate the relationship between unloading and cartilage strain (recovery).

**Figure 1:**
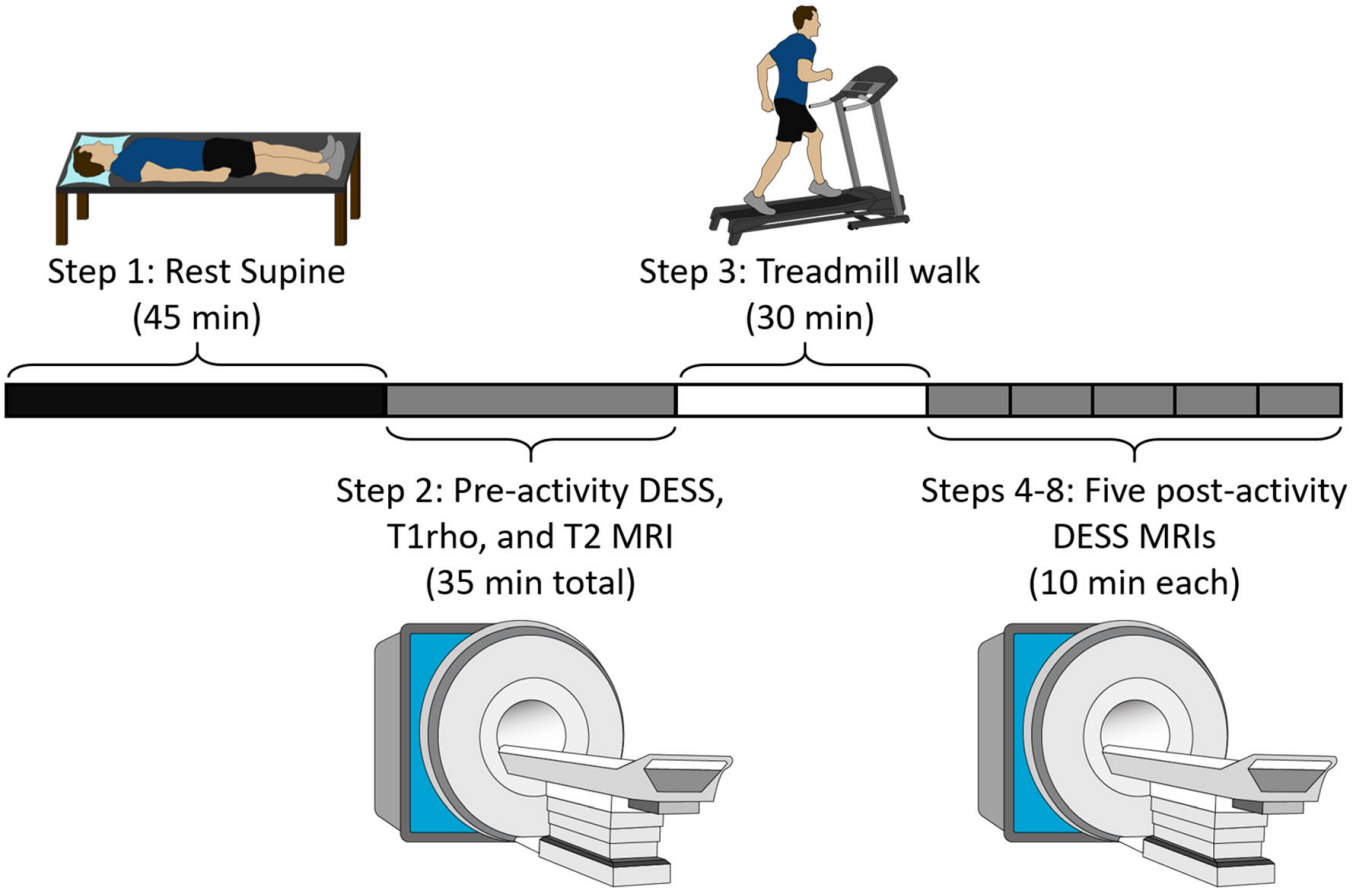
Study timeline for each subject. Figure adapted from Ref. 21

The study consisted of a single visit for each subject (Fig. 1). Subjects were instructed to refrain from strenuous activity the day before the study and arrived at the study center at 7am to minimize cartilage loading prior to the study.^10^,^42^ Upon arrival, subjects rested supine for 45 min to allow for cartilage equilibration.^41^ Then, subjects were transported by wheelchair to the MR scanner where they underwent baseline (pre-activity) MRI using a 3 Tesla scanner (Trio Tim, Siemens, Erlangen, Germany) and an 8-channel knee coil (Invivo Corporation, Gainesville, FL). Specifically, each subject’s dominant knee was imaged while they lay supine with their leg in a relaxed and extended position. Leg dominance was determined by which leg the subject preferred to use to kick a ball.^17^ All MR images were acquired in the sagittal plane, and three different sequences were used to obtain three types of images during the baseline MRI: a double-echo steady-state (DESS) scan, a T1rho scan, and a T2 mapping scan (see Table 1 for scan parameters). Total baseline scan time was approximately 35 min.

**Table 1:** MRI parameters.

Parameter	DESS scan	T1rho scan	T2 mapping scan
Field of view	16 cm × 16 cm	14 cm × 14 cm	16 cm × 16 cm
Matrix size	512 px × 512 px	256 px × 256 px	384 px × 384 px
Slice thickness	1 mm	3 mm	3 mm
Flip angle	25°	15°	180°
Repetition time	17 ms	3500 ms	3500 ms
Echo time(s)	6 ms	5.9 ms	13.8, 27.6, 41.4, 55.2, 69.0, 82.8, 96.6 ms
Spin lock times	–	5, 10, 40, 80 ms at 500 Hz	–
Duration	~ 10 min	~ 13 min	~ 12 min

After the baseline MRI, subjects walked on a treadmill for 30 min in a room adjacent to the MR scanner. This duration was chosen because it induces statistically significant strain within tibiofemoral cartilage.^26^,^36^ Subjects walked at a fixed, comfortable speed normalized to their lower limb length. Normalization was accomplished using the Froude number (Fr),^2^ which is a dimensionless quantity relating walk speed (ν, in m/s), lower limb length (*L*, in m, defined as the vertical distance from the surface of the floor to the greater trochanter, identified by manual palpation), and the gravitational constant (*g* = 9.81, in m/s^2^) according to Eq. 1. Each subject walked at a normalized speed of *Fr* = 0.25 (corresponding to 1.48 ± 0.05 m/s for these subjects, mean ± standard deviation) which represents a comfortable walking pace for adults.^2^,^25^

1 $$v = \sqrt {\left( {Fr} \right)\left( L \right)\left( g \right)} .$$

Immediately after the walking exercise, subjects underwent five consecutive post-activity DESS MRIs to capture the recovery of cartilage thickness after loading, with no wait time between sequences. As each DESS scan lasted approximately 10 min, the post-activity MRIs captured a total period of 50 min after the walking activity. Time between the walk and MRI was 3:36 ± 0:47 min (mean ± standard deviation).

### Data Analysis—DESS Scans

**Figure 2:**
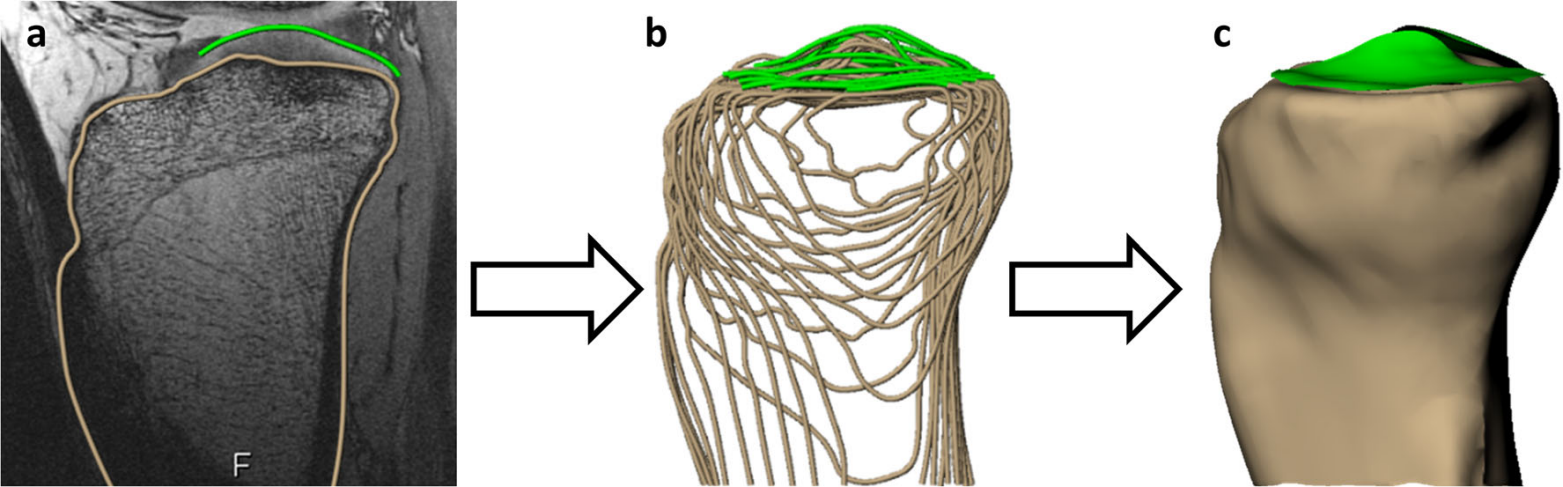
Tibial cartilage thickness measurement procedure, showing (**a**) segmentation of tibial cortex and tibial articular cartilage, (**b**) segmentation stack across several slices, and (**c**) 3D surface mesh model creation

The DESS MR images were used to create virtual three dimensional (3D) knee joint models^8^ from which tibial cartilage thickness was measured (Fig. 2). This technique has been previously shown to measure changes in cartilage thickness with a coefficient of variation of 1%.^10^ Quantifying tibial cartilage thickness involved three steps: (1) manual segmentation by a single investigator of the outer margins of the tibia and the tibial articular cartilage surface in each DESS MR image slice (Fig. 2a) using solid modeling software (Rhinoceros 4.0, Robert McNeel and Associates, Seattle, WA); (2) stacking the segmented contours across slices to create a wireframe model of the joint (Fig. 2b); and (3) generating a 3D surface mesh model from the wireframe models (Fig. 2c) using solid modeling software (Geomagic Studio 11, 3D Systems, Rock Hill, SC). This procedure was carried out for each DESS scan (pre-activity scan and five post-activity scans) to generate a 3D surface mesh model representing the tibia and tibial cartilage at each time point (pre-activity and 10, 20, 30, 40, and 50 min post-activity).

**Figure 3:**
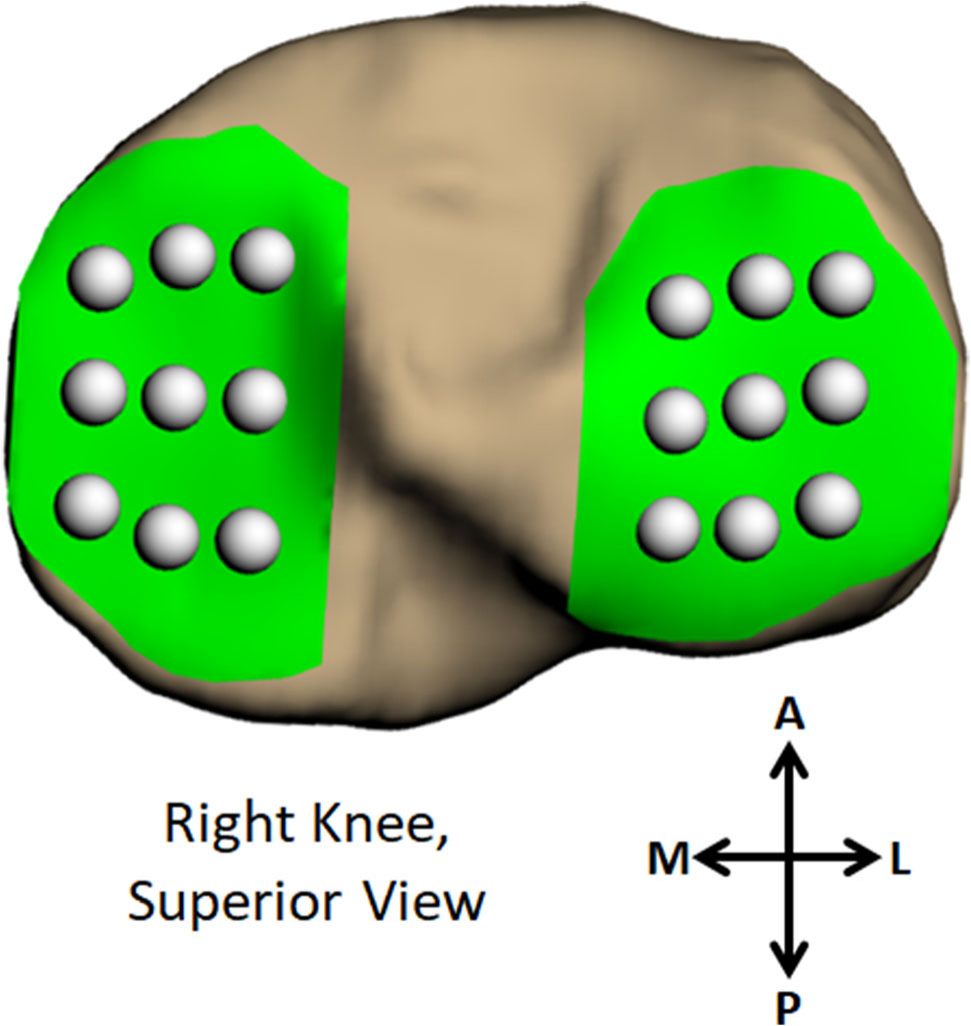
Grid sampling system used to define cartilage thickness in each model (*A* anterior, *P* posterior, *M* medial, *L* lateral)

Next, each post-activity tibia model was aligned with the pre-activity tibia model using an iterative closest-point technique (Geomagic Studio 11, 3D Systems, Rock Hill, SC). This was done in order to sample cartilage thickness at the same locations in the pre- and each post-activity model. To quantify cartilage thickness, 18 sampling points were applied across the surface of the tibial cartilage (9 points arranged in a 3 × 3 grid on the medial and lateral plateaus, respectively; Fig. 3), and custom software (Wolfram Mathematica 9, Wolfram Research, Champaign, IL) was used to measure cartilage thickness at each sampling point.^26^,^36^ Thickness was defined as the distance between each node of the cartilage surface mesh and the nearest node of the tibial surface mesh. The thickness value of all cartilage surface nodes within a 2.5 mm radius were averaged to represent the cartilage thickness at each sampling point. Cartilage strain was calculated for each sampling point as the thickness change from post- to pre-activity, normalized to the pre-activity thickness, expressed as a percent (Eq. 2). All 18 sampling points were averaged to represent the mean cartilage strain per subject at each recovery time point. As our prior work^36^ included measurement of five separate pre-activity scans on a group of ten subjects, each taken on different days, we quantified our technique’s strain error using this data. For each subject, the difference in pre-activity thickness was calculated between the first pre-activity scan and each of the four subsequent pre-activity scans. Each difference was normalized to the first pre-activity thickness and expressed as a percent (Eq. 3), analogous to our calculation of strain (Eq. 2). The mean difference across all four measures per subject and across all ten subjects was 0.2%. As the expected difference in pre-activity thickness between different days is 0% (i.e. a “strain” of 0%), this measurement encompasses both the error resulting from day-to-day differences in cartilage thickness as well as the error in pre- and post-activity model alignment.

2 $$\varepsilon \left( t \right) = \left( {\frac{{h_{\text{post}} - h_{\text{pre}} }}{{h_{\text{pre}} }}} \right) \cdot 100\% ,$$

where $\varepsilon$ = strain, $t$ = time, $h_{pre}$ = pre-activity thickness, and $h_{post}$ = post-activity thickness.

3 $$\Delta h = \left( {\frac{{h_{{{\text{pre}}_{\text{n}} }} - h_{{{\text{pre}}_{ 1} }} }}{{h_{{{\text{pre}}_{ 1} }} }}} \right) \cdot 100\% ,$$

where $\Delta h$ = difference in pre-activity thickness, $h_{{{\text{pre}}_{ 1} }}$ = pre-activity thickness from first pre-activity scan, and $h_{{{\text{pre}}_{\text{n}} }}$ = pre-activity thickness from a subsequent (either second, third, fourth, or fifth) pre-activity scan.

Importantly, *ex vivo* studies using mechanical testing^3^–^5^,^34^,^40^ and a recent *in vivo* MRI study from our group^36^ have demonstrated the viscoelastic behavior of cartilage, commonly described using biphasic theory.^34^ Under biphasic theory, the time constant (Eq. 4c) describes how quickly equilibrium (the asymptote) is reached and is related to the stiffness (*H*_*A*_), thickness (*h*), and permeability (*k*) of the cartilage.^34^ It is often reported as the characteristic time (Eq. 4d),^3^ corresponding to the first term (*n* = 0) in the summation (Eqs. 4a and 4b). As only five time points were quantified in this study, the empirical Kelvin–Voigt viscoelastic two-parameter exponential model^33^ describing recovery (Eq. 5), which represents a first-order approximation of the biphasic equation, was fit to the *in vivo* compressive strain vs. time post-activity (10, 20, 30, 40, and 50 min) data, similar to our previous work.^36^ Thus, the Kelvin–Voigt recovery time constant (1/*B*) of the tissue was calculated as an approximation of the characteristic recovery time. Curve-fitting was carried out using custom software written in MATLAB (version R2018a, Mathworks, Natick, MA). To characterize the cohort of subjects in our study, the mean strain trajectory across time was used for the curve-fit, and was calculated as the mean strain at each post-activity time point across all subjects (*n* = 11). As one subject’s 40 min post-activity scan showed motion artifact, it was not included in the analysis, resulting in *n* = 10 for the 40 min time point. Strains, where reported or graphed, are presented as the mean strain (± 95% confidence interval) at the specified post-activity time point, where a negative sign indicates compression. Finally, to explore relationships between baseline MRI measures (T1rho and T2 relaxation times and cartilage thickness) and the characteristic recovery time, each subject’s strain trajectory was also fit with the Kelvin–Voigt recovery model to calculate the characteristic recovery time on a per-subject basis.

4a $$\varepsilon \left( t \right) = - \frac{\sigma }{{H_{A} }}\left[ {1 - 2\mathop \sum \limits_{n = 0}^{\infty } \frac{1}{M}e^{{ - \left( {\frac{M}{\tau }} \right)t}} } \right],$$

where

4b $$M = \pi^{2} \left( {n + \frac{1}{2}} \right)^{2} ,$$

4c $$\tau = \frac{{h^{2} }}{{H_{A} k}},$$

and

4d $$\tau_{0} = \frac{4}{{\pi^{2} }}\frac{{h^{2} }}{{H_{A} k}},$$

where *ε* = strain, *t* = time, *h* = baseline thickness, $\sigma_{0}$ = applied stress, *H*_*A*_ = aggregate modulus, $\tau$ = time constant, $\tau_{0}$ = characteristic time, and $k$ = permeability.

5 $$\varepsilon \left( t \right) = A\left[ {e^{{ - \left( {Bt} \right)}} } \right],$$

where *ε* = strain, *t* = time, and *A* and *B* are fitted constants.

### Data Analysis—T1rho and T2 Mapping Scans

The T1rho and T2 mapping images were used to calculate T1rho and T2 relaxation times^6^,^20^,^30^ on a per-subject basis using custom software written in MATLAB. First, the cartilage was manually segmented in each slice of the scan corresponding to the first acquired spin lock time (T1rho) or echo time (T2 map) to define the subset of pixels representing cartilage. These same cartilage masks were then applied to the scans from subsequent spin lock times (T1rho) or echo times (T2 map). Next, the signal intensity in each cartilage pixel was extracted for every spin lock time (T1rho) or for every echo time (T2 map). Finally, for each pixel, the signal intensities across time (either spin lock or echo, respectively) were fit with an exponential decay curve (Eqs. 6a or 6b, respectively), where the relaxation time was defined as the decay constant of the exponential curve.^30^ The mean relaxation time across all pixels from all slices was used to represent each subject’s relaxation time (both for T1rho and T2 relaxation times).

6a $$S\left( t \right) = S_{0} \left[ {e^{{ - \left( {\frac{t}{T1rho}} \right)}} } \right],$$

6b $$S\left( t \right) = S_{0} \left[ {e^{{ - \left( {\frac{t}{T2}} \right)}} } \right],$$

where *S*(*t*) = signal intensity, *t* = time—either spin lock (T1rho) or echo (T2 map), $T1rho$ = T1rho relaxation time, and *T2* = T2 relaxation time.

### Statistical Analysis

Statistical modeling was performed in SAS (SAS 9.4, SAS Institute Inc., Cary, NC) with a significance level of *p* < 0.05. Outcome measures (characteristic recovery time, baseline cartilage thickness, baseline T1rho relaxation time, and baseline T2 relaxation time) were assessed for normality using the Shapiro–Wilk test. Two-tailed Pearson correlations were used to test for significant correlations between the outcome measures. Finally, multiple linear regression modeling was performed to determine whether baseline MR-derived measures (cartilage thickness, T1rho relaxation time, T2 relaxation time) were predictive of the characteristic recovery time.

## Results

**Fig. 4:**
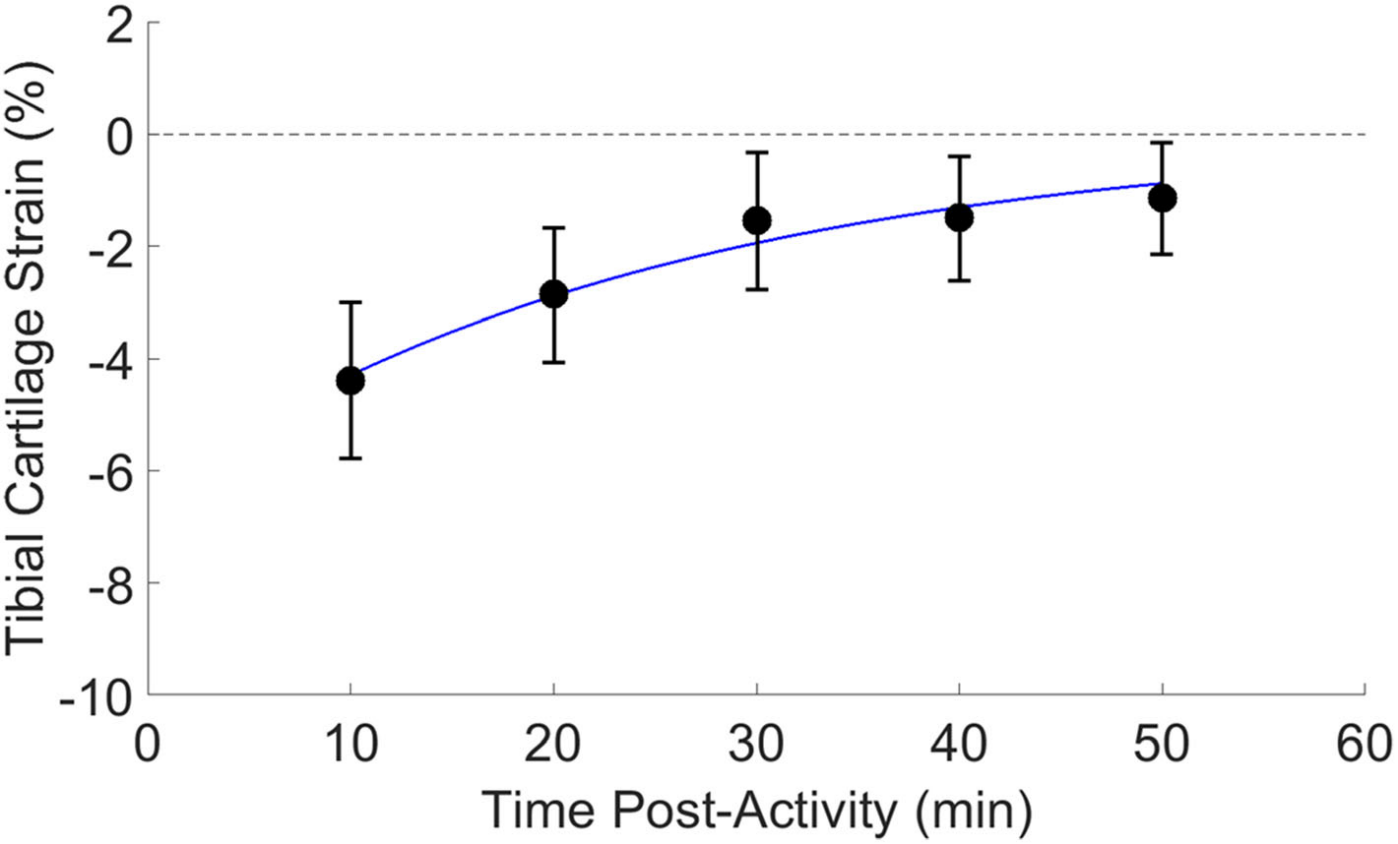
Tibial cartilage strain (%, mean ± 95% confidence interval) across time after activity, including a fit of the empirical Kelvin–Voigt model for recovery. The magnitude of cartilage strain decreased with time post-activity

As expected, the magnitude of cartilage strain incurred by the 30 min walk decreased with time after activity, indicative of the recovery of cartilage thickness with time after activity. Specifically, at 10 min post-activity, the mean strain was − 4 ± 1% (± 95% confidence interval), while at 50 min post-activity, it had recovered to − 1 ± 1% (Fig. 4). Indeed, the mean strain trajectory across time (calculated as the mean strain value across subjects at each post-activity time point) was well-fit by the empirical Kelvin–Voigt exponential model for recovery (Fig. 4), with a characteristic recovery time of 25.2 min (root mean square error, RMSE = 0.24; RMSE of a line fit to these data = 0.46).

The correlation analysis and multiple linear regression model predicting the characteristic recovery time as a function of baseline measures (cartilage thickness, T1rho relaxation time, T2 relaxation time) was based on a subset of our cohort (8 subjects), as three subjects did not complete T2 mapping scans. No significant correlations between any of the outcome measures were found, and the multiple linear regression model did not find any of the baseline measures to be significant predictors of the characteristic recovery time (Table 2).

**Table 2:** Parameter estimates from a multiple linear regression predicting the characteristic recovery time.

Variable	Parameter estimate	Standard error	*p* value
Baseline thickness (mm)	37.46	24.61	0.20
Mean T1rho relaxation time (ms)	2.31	1.33	0.16
Mean T2 relaxation time (ms)	0.79	2.54	0.77

## Discussion

As the diagnosis of OA depends on features that are typically not present until advanced stages of the disease (including radiographic findings and pain),^32^ assessment of changes that can be observed at earlier stages of disease progression is important for improved identification, prevention, and treatment of OA. Because cartilage’s mechanical response and composition both change during OA progression,^24^ their measurement *in vivo* may enable earlier detection of OA. As qMRI techniques allow for the noninvasive assessment of cartilage composition *in vivo*, we sought here to propose a noninvasive MRI methodology for the assessment of cartilage mechanical response *in vivo*. As such, in this study we measured the *in vivo* recovery response of tibial cartilage to 30 min of walking using MRI in a cohort of relatively young, adult human subjects with asymptomatic cartilage. This enabled us to quantify the characteristic recovery time, a mechanical property of cartilage tissue, *in vivo*. In addition, we measured baseline values of cartilage thickness, T1rho relaxation time, and T2 relaxation time in our cohort, in order to examine predictive relationships between these baseline measures and the characteristic recovery time.

Through the use of our noninvasive MRI methodology evaluating cartilage recovery, we found the *in vivo* characteristic recovery time of our cohort to be 25.2 min. This value is consistent with human cartilage characteristic recovery times reported in the literature,^1^,^3^ obtained through *ex vivo* mechanical testing (Table 3). Using the reported literature values of mean explant height, stiffness, and permeability from two studies of creep in *ex vivo* human cartilage,^1^,^3^ we calculated the corresponding characteristic creep times *via* Eq. 4d^3^,^34^ to be 26.8 min for tibial plateau cartilage^1^ and 17.7 min for patellar cartilage.^3^ These are similar to the characteristic recovery time measured in the present *in vivo* study (25.2 min for tibial plateau cartilage). While future work is needed to understand how the characteristic recovery time changes with OA, the consistency between our *in vivo* results and *ex vivo* literature findings validates our novel MRI approach, and reveals the utility of the characteristic recovery time as an *in vivo* metric reflecting cartilage’s mechanical function.

**Table 3:** Literature values of human cartilage creep characteristic time.

References	Cartilage location	Sample height (*h*, mm)	Stiffness (*H*_*A*_, MPa)	Permeability (*k*, *x* 10^−15^ m^4^/Ns)	Characteristic time ($\tau_{0}$, min)
Akizuki *et al*.^1^	Tibial Plateau	2.27	0.65	2.00	26.8
Armstrong and Mow^3^	Patella	3.12	0.79	4.70	17.7

Additionally, our observations of cartilage’s recovery trajectory are comparable to other observations in the literature. For example, a recent MRI study^44^ found that in response to 30 min of running, femoral and tibial cartilage volume of control subjects recovered to within 1% of baseline after 45 min. Likewise, another MRI study investigating a running activity found that tibial cartilage volume recovered to within 1% of baseline after 60 min.^23^ In the current study, tibial cartilage thickness also recovered to within 1% of baseline after a similar amount of time (strain after 50 min of recovery, mean ± 95% confidence interval: − 1 ± 1%). Finally, the *in vivo* recovery response measured here is similar to our prior work investigating the *in vivo* loading response, which was also well-fit by the Kelvin–Voigt model for creep.^36^ The similarity between the *in vivo* creep and recovery strain trajectories is in line with our *ex vivo* results, in which comparable mechanical property measurements resulted from assessing either the loading or the unloading response.^12^

Moreover, the characteristic recovery time was not significantly correlated with the baseline MRI metrics measured here (T1rho relaxation time, T2 relaxation time, cartilage thickness), nor were these metrics significant predictors of the characteristic recovery time. This might suggest that the characteristic recovery time contributes independent information about the mechanical state of the cartilage not captured by the other MRI metrics measured in this study. This further underscores the characteristic recovery time’s value as an indicator of cartilage’s mechanical function, and illustrates the potential importance of its measurement in addition to baseline qMRI measures of cartilage composition for the noninvasive assessment of cartilage health. However, this study analyzed only relatively young and asymptomatic volunteers, and may therefore have been underpowered to detect relationships between the characteristic recovery time and baseline measures with the current sample size. As T1rho and T2 relaxation times change with OA progression,^30^ these baseline measures may be more indicative of mechanical response if analyzed when a larger variation in cartilage health is assessed. On the other hand, recent work investigating changes in stiffness and histology in murine cartilage after surgical destabilization of the medial meniscus found that significant changes in stiffness were apparent sooner after the surgery (within a week) than were histological changes as assessed by Mankin grading (within four to 8 weeks).^13^ This supports the hypothesis that mechanical changes may be one of the earliest changes occurring in the progression of OA. Furthermore, our results suggest that baseline qMRI may not fully capture information about cartilage mechanical properties such as the characteristic recovery time. Overall, as cartilage mechanical response changes as cartilage health changes,^20^,^24^,^37^,^39^ the characteristic recovery time presented here may represent another useful indicator of *in vivo* cartilage health that is important to quantify in addition to baseline measures of composition and morphology. However, future studies, including assessment of characteristic recovery time in OA patients and those at risk of OA (such as with obesity or joint injury), are required to fully understand the value of the characteristic recovery time as an indicator of cartilage health.

Overall, in this work we developed a novel methodology for measuring *in vivo* cartilage mechanical properties, by assessing the recovery response after loading and quantifying the characteristic recovery time *in vivo*. The results of this study show that comparable characteristic times (mechanical properties) are measured using our *in vivo* MRI approach as they are using literature values from *ex vivo* mechanical testing, supporting the validity of our methodology. Additionally, because cartilage mechanical properties change with OA progression,^20^,^24^,^37^,^39^ the characteristic recovery time may represent a potential noninvasive MRI marker of cartilage health, as it represents a combination of these mechanical properties (by Eq. 4d). Therefore, we propose the characteristic recovery time to be a new, noninvasive metric describing the mechanical function of cartilage. As such, the results presented here quantify normative values of the *in vivo* characteristic time of asymptomatic tibial plateau cartilage, against which future values of OA cartilage can be compared. Future studies investigating how the characteristic recovery time changes with OA severity are warranted to assess its value as a marker of early-stage OA and its relationship to cartilage health.

## References

[1] Akizuki S, Mow V, Lai W, Pita J, Howell D (1986). Topographical variations of the biphasic indentation properties of human tibial plateau cartilage. Trans. Orthop. Res. Soc.

[2] Alexander R, Jayes A (1983). A dynamic similarity hypothesis for the gaits of quadrupedal mammals. J. Zool..

[3] Armstrong C, Mow V (1982). Variations in the intrinsic mechanical properties of human articular cartilage with age, degeneration, and water content. J. Bone Joint Surg. Am..

[4] Ateshian G, Warden W, Kim J, Grelsamer R, Mow V (1997). Finite deformation biphasic material properties of bovine articular cartilage from confined compression experiments. J. Biomech..

[5] Athanasiou K, Rosenwasser M, Buckwalter J, Malinin T, Mow V (1991). Interspecies comparisons of in situ intrinsic mechanical properties of distal femoral cartilage. J. Orthop. Res..

[6] Borthakur A, Wheaton A, Charagundla SR, Shapiro EM, Regatte RR, Akella SV, Kneeland JB, Reddy R (2003). Three-dimensional T1ρ-weighted MRI at 1.5 Tesla. J. Magn. Reson. Imaging.

[7] Buschmann MD, Gluzband YA, Grodzinsky AJ, Hunziker EB (1995). Mechanical compression modulates matrix biosynthesis in chondrocyte/agarose culture. J. Cell Sci..

[8] Carter TE, Taylor KA, Spritzer CE, Utturkar GM, Taylor DC, Moorman CT, Garrett WE, Guilak F, McNulty AL, DeFrate LE (2015). In vivo cartilage strain increases following medial meniscal tear and correlates with synovial fluid matrix metalloproteinase activity. J. Biomech..

[9] Chan DD, Cai L, Butz KD, Trippel SB, Nauman EA, Neu CP (2016). In vivo articular cartilage deformation: noninvasive quantification of intratissue strain during joint contact in the human knee. Sci. Rep..

[10] Coleman JL, Widmyer MR, Leddy HA, Utturkar GM, Spritzer CE, Moorman CT, Guilak F, DeFrate LE (2013). Diurnal variations in articular cartilage thickness and strain in the human knee. J. Biomech..

[11] Collins AT, Hatcher C, Kim S, Ziemian S, Spritzer C, Guilak F, DeFrate L, McNulty A (2018). Selective enzymatic digestion of proteoglycans and collagens alters cartilage T1rho and T2 relaxation times. Ann. Biomed. Eng..

[12] Cutcliffe HC, DeFrate LE (2020). Comparison of cartilage mechanical properties measured during creep and recovery. Sci. Rep..

[13] Doyran B, Tong W, Li Q, Jia H, Zhang X, Chen C, Enomoto-Iwamoto M, Lu XL, Qin L, Han L (2017). Nanoindentation modulus of murine cartilage: a sensitive indicator of the initiation and progression of post-traumatic osteoarthritis. Osteoarthr. Cartil..

[14] Eckstein F, Lemberger B, Gratzke C, Hudelmaier M, Glaser C, Englmeier K, Reiser M (2005). In vivo cartilage deformation after different types of activity and its dependence on physical training status. Ann. Rheum. Dis..

[15] Eckstein F, Tieschky M, Faber S, Englmeier K-H, Reiser M (1999). Functional analysis of articular cartilage deformation, recovery, and fluid flow following dynamic exercise in vivo. Anat. Embryol..

[16] Eckstein F, Tieschky M, Faber SC, Haubner M, Kolem H, Englmeier K-H, Reiser M (1998). Effect of physical exercise on cartilage volume and thickness in vivo: MR imaging study. Radiology.

[17] Gabbard C, Hart S (1996). A question of foot dominance. J. Gen. Psychol..

[18] Guermazi A, Alizai H, Crema M, Trattnig S, Regatte R, Roemer F (2015). Compositional MRI techniques for evaluation of cartilage degeneration in osteoarthritis. Osteoarthr. Cartil..

[19] Guilak F (2011). Biomechanical factors in osteoarthritis. Best Pract. Res. Clin. Rheumatol..

[20] Hatcher CC, Collins AT, Kim SY, Michel LC, Mostertz WC, Ziemian SN, Spritzer CE, Guilak F, DeFrate LE, McNulty AL (2017). Relationship between T1rho magnetic resonance imaging, synovial fluid biomarkers, and the biochemical and biomechanical properties of cartilage. J. Biomech..

[21] Heckelman LN, Smith WAR, Riofrio AD, Vinson EN, Collins AT, Gwynn OR, Utturkar GM, Goode AP, Spritzer CE, DeFrate LE (2020). Quantifying the biochemical state of knee cartilage in response to running using T1rho magnetic resonance imaging. Sci. Rep..

[22] Hollander A, Pidoux I, Reiner A, Rorabeck C, Bourne R, Poole AR (1995). Damage to type II collagen in aging and osteoarthritis starts at the articular surface, originates around chondrocytes, and extends into the cartilage with progressive degeneration. J. Clin. Invest..

[23] Kessler MA, Glaser C, Tittel S, Reiser M, Imhoff AB (2008). Recovery of the menisci and articular cartilage of runners after cessation of exercise additional aspects of in vivo investigation based on 3-dimensional magnetic resonance imaging. Am. J. Sports Med..

[24] Knecht S, Vanwanseele B, Stüssi E (2006). A review on the mechanical quality of articular cartilage-implications for the diagnosis of osteoarthritis. Clin. Biomech..

[25] Kramer PA, Sylvester AD (2012). Humans, geometric similarity and the Froude number: is ‘‘reasonably close’’really close enough?. Biol Open.

[26] Lad NK, Liu B, Ganapathy PK, Utturkar GM, Sutter EG, Moorman CT, Garrett WE, Spritzer CE, DeFrate LE (2016). Effect of normal gait on in vivo tibiofemoral cartilage strains. J. Biomech..

[27] Larsson T, Aspden RM, Heinegård D (1991). Effects of mechanical load on cartilage matrix biosynthesis in vitro. Matrix.

[28] Lee DA, Bader DL (1997). Compressive strains at physiological frequencies influence the metabolism of chondrocytes seeded in agarose. J. Orthop. Res..

[29] Li X, Cheng J, Lin K, Saadat E, Bolbos RI, Jobke B, Ries MD, Horvai A, Link TM, Majumdar S (2011). Quantitative MRI using T1ρ and T2 in human osteoarthritic cartilage specimens: correlation with biochemical measurements and histology. Magn. Reson. Imaging.

[30] Li X, Ma B, Thomas L, Castillo D, Blumenkrantz G, Lozano J, Carballido-Gamio J, Ries M, Majumdar S (2007). In vivo T1Rho and T2 mapping of articular cartilage in osteoarthritis of the knee using 3 tesla MRI. Osteoarthr. Cartil..

[31] Li X, Pai A, Blumenkrantz G, Carballido-Gamio J, Link T, Ma B, Ries M, Majumdar S (2009). Spatial distribution and relationship of T1ρ and T2 relaxation times in knee cartilage with osteoarthritis. Magn. Reson. Med..

[32] Lorenz H, Richter W (2006). Osteoarthritis: cellular and molecular changes in degenerating cartilage. Prog. Histochem. Cytochem..

[33] Mow VC, Huiskes R (2005). Basic Orthopaedic Biomechanics & Mechano-biology.

[34] Mow VC, Kuei S, Lai WM, Armstrong CG (1980). Biphasic creep and stress relaxation of articular cartilage in compression: theory and experiments. J. Biomech. Eng..

[35] Nishioka H, Hirose J, Nakamura E, Oniki Y, Takada K, Yamashita Y, Mizuta H (2012). T1ρ and T2 mapping reveal the in vivo extracellular matrix of articular cartilage. J. Magn. Reson. Imaging.

[36] Paranjape CS, Cutcliffe HC, Grambow SC, Utturkar GM, Collins AT, Garrett WE, Spritzer CE, DeFrate LE (2019). A New Stress Test for Knee Joint Cartilage. Scientific Reports.

[37] Rivers P, Rosenwasser M, Mow V, Pawluk R, Strauch R, Sugalski M, Ateshian G (2000). Osteoarthritic changes in the biochemical composition of thumb carpometacarpal joint cartilage and correlation with biomechanical properties. J. Hand Surg..

[38] Sah RLY, Kim YJ, Doong JYH, Grodzinsky AJ, Plass AH, Sandy JD (1989). Biosynthetic response of cartilage explants to dynamic compression. J. Orthop. Res..

[39] Setton L, Mow V, Müller F, Pita J, Howell D (1994). Mechanical properties of canine articular cartilage are significantly altered following transection of the anterior cruciate ligament. J. Orthop. Res..

[40] Soltz MA, Ateshian GA (1998). Experimental verification and theoretical prediction of cartilage interstitial fluid pressurization at an impermeable contact interface in confined compression. J. Biomech..

[41] Sutter EG, Widmyer MR, Utturkar GM, Spritzer CE, Garrett WE, DeFrate LE (2015). In vivo measurement of localized tibiofemoral cartilage strains in response to dynamic activity. Am. J. Sports Med..

[42] Taylor KA, Collins AT, Heckelman LN, Kim SY, Utturkar GM, Spritzer CE, Garrett WE, DeFrate LE (2018). Activities of daily living influence tibial cartilage T1rho relaxation times. J. Biomech..

[43] Thompson R, Oegema T (1979). Metabolic activity of articular cartilage in osteoarthritis. An in vitro study. J. Bone Joint Surg. Am..

[44] Van Ginckel A, Verdonk P, Victor J, Witvrouw E (2013). Cartilage status in relation to return to sports after anterior cruciate ligament reconstruction. Am. J. Sports Med..

[45] Wayne JS, Kraft KA, Shields KJ, Yin C, Owen JR, Disler DG (2003). MR imaging of normal and matrix-depleted cartilage: correlation with biomechanical function and biochemical composition. Radiology.

[46] WHO Obesity: Preventing and Managing the Global Epidemic. World Health Organization, 2000. 11234459

